# Flight of the COVID-19 patient: experience with a Wuhan evacuee: a case report

**DOI:** 10.1186/s13256-020-02396-8

**Published:** 2020-06-11

**Authors:** Sandeep Segar, Daniel Bouland, Francesca Torriani, Kevin Kwak, Deepak Asudani, Randy Taplitz, Vineet Gupta

**Affiliations:** 1 Division of Hospital Medicine, University of California San Diego Health, 200 West Arbor Drive, MC 8485, San Diego, CA 92103 USA; 2 Department of Internal Medicine, University of California San Diego (UCSD) Health, 200 West Arbor Drive, San Diego, CA 92103 USA; 3 Division of Infectious Diseases and Global Public Health, 200 West Arbor Drive, San Diego, CA 92103 USA

**Keywords:** 2019 novel coronavirus, SARS-CoV-2, COVID-19, Severe acute respiratory syndrome coronavirus 2, Remdesivir, Quarantine

## Abstract

**Background:**

Coronavirus disease 2019, caused by severe acute respiratory syndrome coronavirus 2, was declared a global pandemic by the World Health Organization in March 2020.

**Case presentation:**

We report a case of a 51-year-old Chinese woman who was evacuated from Wuhan, China and diagnosed with coronavirus disease 2019 infection at a Southern California quarantine facility. Her clinical course was notable for high fevers, night sweats, productive cough, transient leukopenia, lymphopenia, thrombocytopenia, and transaminitis. Evolving hypoxia and infiltrates on chest imaging warranted the trial of an investigational antiviral drug - remdesivir. Our patient recovered and was discharged after 2 weeks of hospitalization.

**Conclusions:**

This case highlights our patient’s clinical course, including diagnostic work-up, medical management, and challenges in defining non-infectivity in a relatively unknown disease.

## Introduction

A cluster of unexplained pneumonia cases linked to the Huanan seafood market in Wuhan, China was first reported on December 31, 2019 [1]. After testing negative for common respiratory viruses, these patients tested positive for a novel coronavirus - severe acute respiratory syndrome-related coronavirus 2 (SARS-CoV-2), which is the cause of coronavirus disease 2019 (COVID-19) [2, 3]. Some of these initial patients demonstrated hypoxemia, ground glass opacification on chest imaging, abnormal laboratory results - low white blood cell (WBC) count, low absolute lymphocyte count (ALC), low platelet count, elevated liver enzymes, and elevated creatinine [2].

COVID-19 was declared a global pandemic by the World Health Organization with over 750,000 confirmed cases in over 200 countries and territories as of March 31, 2020 [4]. We report the case of an early patient with COVID-19 who was evacuated from Wuhan, China and developed signs of infection at a Southern California quarantine facility. This case highlights our patient’s clinical course, including relevant history, diagnostic work-up, medical management, and challenges in defining non-infectivity.

## Case presentation

A 51-year-old Chinese woman with no significant past medical history presented to our institution with a 1-day history of fevers, chills, sweats, nonproductive cough from a Southern California quarantine facility. She had worked as a nurse in an outpatient medicine clinic in Wuhan, China, where she cared for patients with upper respiratory tract symptoms, but without confirmed diagnoses of COVID-19. While caring for these patients, she reported wearing a standard surgical mask and gloves. Three weeks prior to hospital admission, upon becoming aware of a rapidly spreading pulmonary infection within her community, our patient took leave from work and self-isolated herself in her apartment with her husband and grandson, neither of whom had signs of infection. She had no direct exposure to the Huanan seafood market.

Four days prior to hospital admission, she and her grandson were evacuated on a flight from the Wuhan International Airport. Late the next day, she arrived at a Southern California government facility for an intended 14-day quarantine. One day prior to admission, our patient developed a nonproductive cough, fever, chills and sweats. The same day, nasopharyngeal (NP) and oropharyngeal (OP) swabs for COVID-19 using reverse transcription polymerase chain reaction (RT-PCR) were sent from the quarantine facility to the US Centers for Disease Control and Prevention (CDC) laboratory per recommended guidelines (Appendix).

The following day, she was admitted to our institution and was placed in contact, droplet, and airborne isolation precautions per CDC recommendations. At admission, her temperature was 38.4 °C, blood pressure was 101/69 mm Hg, heart rate was 84 beats per minute, respiratory rate was 17 breaths per minute, and oxygen saturation was 96% on room air. A physical examination including cardiopulmonary evaluation was unremarkable. Laboratory studies were notable for WBC 3600 per mm^3^ (range 4100–10,400/mm^3^), absolute neutrophil count (ANC) 2100 per mm^3^, ALC 1000 per mm^3^, and platelet count 121,000 per mm^3^. Other test results including liver function tests, coagulation studies, procalcitonin, and urinalysis were unremarkable. A NP swab using Reverse transcription polymerase chain reaction (RT-PCR) was negative for usual viral pathogens, including influenza A/B, respiratory syncytial virus, human rhinovirus/enterovirus, human metapneumovirus, parainfluenza, and four common coronavirus strains previously known to cause human illness (229E, HKU1, NL63, and OC43). A chest X-ray demonstrated clear lung fields bilaterally without consolidation or effusion (Fig. 2a). Serial NP and OP swabs were tested every other day per CDC guidance to evaluate clearance of infection. No antibiotics were given to our patient. NP and OP swabs from the day prior to admission returned positive for COVID-19, and our patient became the 14th confirmed case in the United States.

**Fig. 1:**
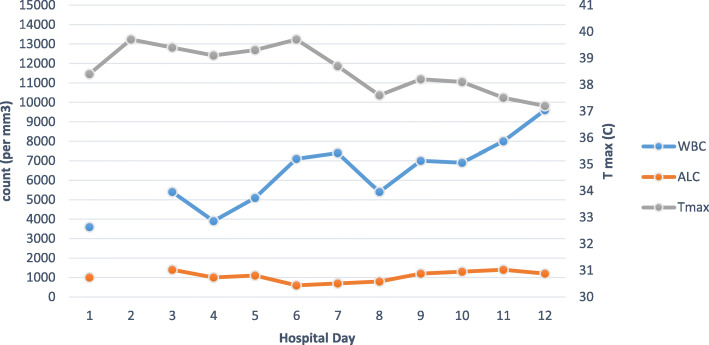
Maximum temperature (Tmax), white blood cell (WBC) count, and absolute lymphocyte count (ALC) trend during hospitalization

**Fig. 2:**
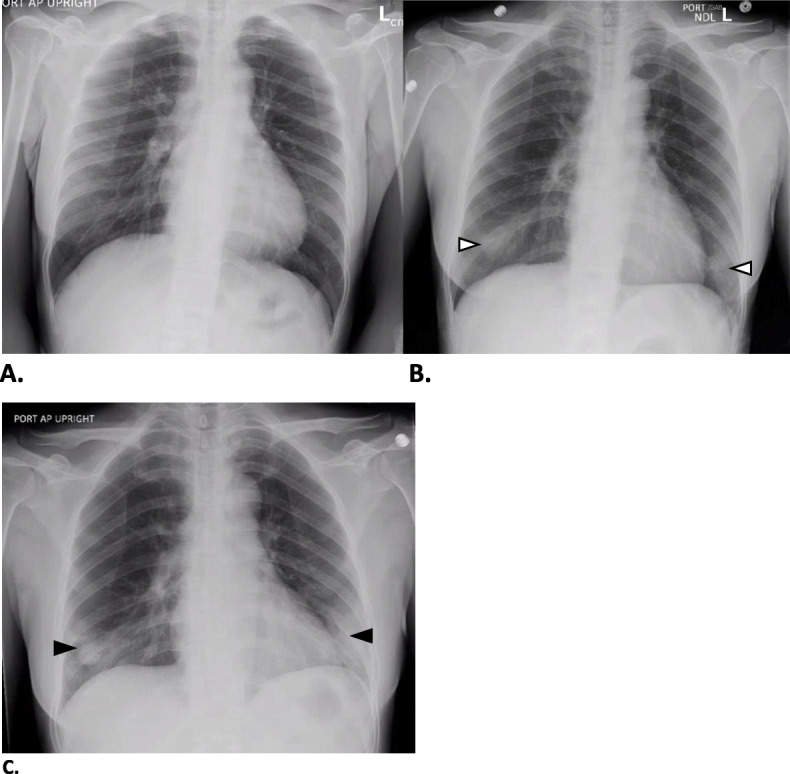
Chest X-ray from (**a**) hospital day 1, (**b**) day 3, and (**c**) day 6 showing serial worsening bibasilar infiltrates, White arrows: developing infiltrates, and Black arrows: worsening infiltrates

**Fig. 3:**
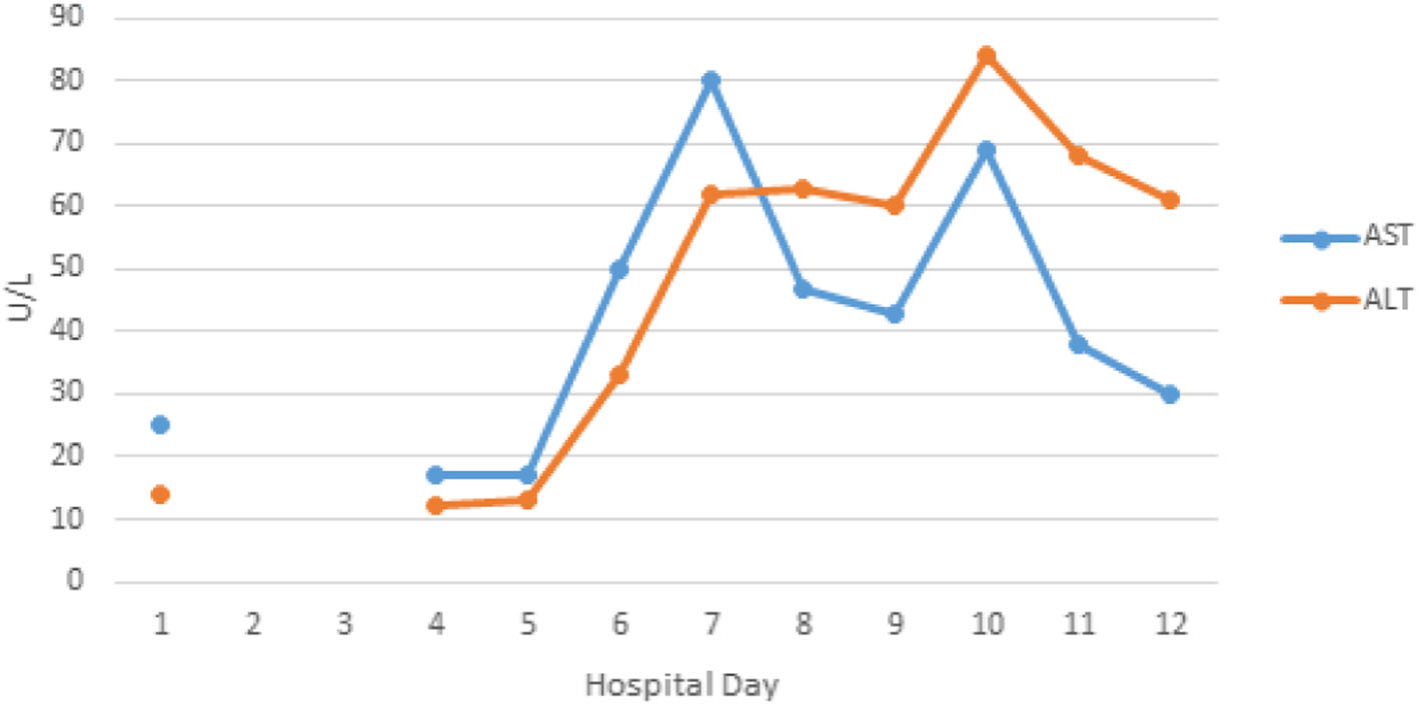
Liver function test (aspartate aminotransferase [AST] and alanine aminotransferase [ALT]) trend during hospitalization

During days 1–6 of hospitalization, she experienced daily fevers, chills, drenching night sweats, and a nonproductive cough. By day 3, she had dyspnea with minimal exertion, exacerbated by coughing fits. She also had diminished appetite with occasional nausea. She denied abdominal pain, diarrhea, or dysuria. Between days 3 and 6, her maximum temperature (Tmax) ranged from 39.0 °C to 39.7 °C (Fig. 1). Her blood pressure remained in low normal range. A pulmonary examination demonstrated bibasilar crackles without labored breathing. On hospital day 3, her chest X-ray demonstrated new findings of bilateral lower lobe reticular opacities (Fig. 2b). Her platelets reached a nadir of 101,000 per mm^3^ on day 4, before improving. Her WBC count improved to 5100 per mm^3^ on hospital day 5, however, ALC decreased to 600 per mm^3^ on day 6, before recovering (Fig. 1). Liver enzymes remained normal until day 6 when aspartate aminotransferase (AST) rose to 50 U/L (Fig. 3). A transthoracic echocardiogram was unremarkable. Her chest X-ray on day 6 showed worsened bibasilar reticular opacification (Fig. 2c). Her blood cultures remained negative during hospitalization. A Quantiferon-TB test resulted negative. Treatment was largely supportive during this time, comprising intravenous hydration and antipyretic therapy (acetaminophen < 4 g daily, ibuprofen, and axillary ice packs).

On day 6 of hospitalization, due to persistent high fevers, relative hypoxemia, evolving chest X-ray findings, and mildly abnormal liver function, our patient was initiated on compassionate use remdesivir 200 mg intravenous on day 1 followed by 100 mg daily. Remdesivir, an investigational antiviral for COVID-19, was previously used in Ebola virus disease, severe acute respiratory syndrome coronavirus (SARS-CoV) and Middle East respiratory syndrome coronavirus (MERS-CoV) [5]. Her fever curve improved on day 8 (Fig. 1). Our patient continued to have mild dyspnea, nausea, poor appetite, and fatigue. Her cough became productive of scant white sputum. Alanine aminotransferase (ALT) peaked at 84 U/L on day 10, before trending down (Fig. 3). As of hospital day 11, she remained afebrile off antipyretics. Her oxygen saturations improved to 97% on ambient air. Her dyspnea and auscultatory rales resolved, however, a mild productive cough persisted.

On hospital day 9, the CDC reported serial NP and OP swabs from her entire hospitalization to be negative (Table 1). This included samples at the height of illness severity (days 1–7). Due to concerns with the adequacy of negative OP and NP testing in defining infection clearance, sputum PCR for COVID-19 on days 10 and 11 were obtained. Surprisingly, both sputum samples returned positive (Table 1). Given the possibility that her productive cough harbored transmissible live virus, our patient was discharged on day 15 to a government medical facility for continued isolation until the resolution of all symptoms. At the time of transfer, she remained afebrile and asymptomatic apart from a mild productive cough. Our patient returned home after 19 days at the government isolation facility after resolution of her cough. She remained non-symptomatic at home when last followed up.

**Table 1:** Infection surveillance tests during hospitalization

Test	Day 0^b^	Day 1	Day 3	Day 5	Day 7	Day 8	Day 10	Day 11
NP swab^a^	Pos	Neg	Neg	Neg	Neg	Neg		
OP swab^a^	Pos	Neg	Neg	Neg	Neg	Neg		
Sputum^a^							Pos	Pos

*Neg* negative, *NP* nasopharyngeal, *OP* oropharyngeal, *Pos* positive

^a^See Appendix

^b^Day 0 correlates with day of illness onset (tests done at quarantine facility), day 1 is initial day of hospitalization

## Discussion

Our case helps offer insight into the clinical course of COVID-19 patients. Coronavirus disease is thought to be transmitted from person to person by respiratory droplets and direct contact [6]. Despite working as an outpatient nurse, it is less likely that she contracted COVID-19 at the workplace as symptom onset was 3 weeks after her last known patient contact. The incubation period of COVID-19 is estimated to be between 2 and 14 days [7–9]. Alternatively, she may have been exposed during her self-imposed home isolation in Wuhan, however her apparent contacts, including her husband and grandson, were asymptomatic. Her grandson later tested negative for COVID-19. There is a likelihood that she was infected during transit to the quarantine facility at either the Wuhan International Airport or on her flight to the United States. Exposure at the quarantine facility seems less likely as that would reflect an incubation period of only a day.

Similar to the first documented cohort of COVID-19 patients linked to the Huanan seafood market, our patient had leukopenia and thrombocytopenia, both of which recovered during the course of her illness [2]. She notably developed lymphopenia on hospital day 6, which also recovered. The rise in liver enzymes was more likely related to the disease course of COVID-19 than to drug-induced liver injury from remdesivir, as enzyme levels down trended to normal during daily antiviral infusions. No other clear implicating medications causing liver injury were utilized. Relative hypoxemia was noted by oxygen saturation, however, our patient never required supplemental oxygen. Chest imaging findings were also significant for infiltrates. In contrast to the reported cohort, [2] renal function in this patient remained normal during the disease course.

The clinical improvement in our patient coincided with the initiation of remdesivir on day 6 of hospitalization. However, it is unclear if this improvement can be attributed to the drug and/or to the natural course of COVID-19 infection. Ongoing investigational trials will help establish the efficacy of remdesivir and other antivirals in COVID-19 management [10–12].

CDC-guided NP and OP samples of our patient from hospital day 1 onward all tested negative for COVID-19 (Table 1). Although our patient was clinically ill enough to warrant continued hospitalization and initiation of the antiviral remdesivir; she would have met laboratory criteria to clear isolation precautions by hospital day 3 with two negative NP and OP specimens on two separate days. During hospital days 1–7, her high fevers and infectious signs in the setting of NP and OP negativity question whether our patient was infective at the time or whether these were manifestations of a prolonged, excessive cytokine and chemokine response that has been documented in other highly pathogenic coronaviruses including SARS-CoV and MERS-CoV [13].

Our patient’s positive sputum RT-PCR result for COVID-19 likely represented inactive viral shedding in the lower respiratory tract rather than active contagious virus, given she had dramatic clinical improvement by hospital day 11. In the 2003 SARS-CoV outbreak, viral shedding was detected in the respiratory, gastrointestinal, and urinary tracts for many weeks after onset of illness, while active transmission of infection was not noted after 2 weeks [14]. The pragmatic correlation of SARS-CoV-2 detection by RT-PCR in sputum, NP, and OP samples with infectivity is warranted to explore similar trends for COVID-19. Further research aimed at the understanding of transmission dynamics will help to guide screening practices and support clearance of infectivity.

## Conclusions

Our experience with one of the earliest cases of COVID-19 in the United States offers insight into the pertinent clinical and laboratory findings of this novel disease entity. Use of remdesivir without any overt side effects in our patient, supports the ongoing clinical trials as a candidate therapeutic agent in COVID-19. In addition, the case highlights possible areas for improvement in diagnosing coronavirus disease and robust protocols in establishing clearance of infection to facilitate removal of isolation precautions and safe return of patients to the community. These topics are pertinent for controlling disease spread and promoting public safety.

## Data Availability

Not applicable.

## Appendix

CDC guidelines for clinical specimen for diagnostic testing of COVID-19. For nasopharyngeal and oropharyngeal swabs, synthetic fiber swabs with plastic shafts were used and immediately placed into sterile tubes containing 2–3 mL of viral transport media; each nasopharyngeal and oropharyngeal swab was kept in a separate vial. The specimens were refrigerated at 2–8^o^ C and shipped overnight to the CDC on ice packs. For sputum samples, our patient expectorated directly into a sterile, leak-proof, screw-cap sputum collection cup, which was then refrigerated at 2–8^o^ C and shipped overnight to the CDC. A RT-PCR assay, which was developed from publicly released virus sequence, was utilized in testing the clinical specimens.

## Abbreviations

ALC: Absolute lymphocyte count
ALT: Alanine aminotransferase
ANC: Absolute neutrophil count
AST: Aspartate aminotransferase
CDC: US Centers for Disease Control and Prevention
COVID-19: Coronavirus disease 2019
MERS-CoV: Middle East respiratory syndrome coronavirus
NP: Nasopharyngeal
OP: Oropharyngeal
RT-PCR: Reverse transcription polymerase chain reaction
SARS-CoV: Severe acute respiratory syndrome coronavirus
SARS-CoV-2: Severe acute respiratory syndrome-related coronavirus 2
Tmax: Maximum temperature
WBC: White blood cell
